# Unusual case of blastoid cells with bilobed nuclei: Cottage loaves, sliding plates, and butterflies

**DOI:** 10.1002/jha2.291

**Published:** 2021-09-28

**Authors:** Emad Ababneh, Alex V. Mejia Garcia, Megan O. Nakashima

**Affiliations:** ^1^ Department of Laboratory Medicine Cleveland Clinic Cleveland Ohio USA; ^2^ Department of Hematology and Medical Oncology Cleveland Clinic Cleveland Ohio USA


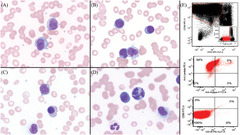


A 66‐year‐old man presented with abdominal pain, fatigue, dyspnea, leukocytosis (40×10^9^/L), and anemia (7 g/dL). Laboratory studies showed kidney injury (creatinine: 2.2 mg/dL), hyperuricemia (uric acid10 mg/dL), and IgG lambda paraprotein (4.54 g/dL). Eighty‐five percent of nucleated cells in peripheral blood were variably sized atypical cells with scant to moderate amounts of basophilic cytoplasm and eccentric nuclei with a perinuclear Golgi hof in most cells (A and B). Some larger cells showed bilobed nuclei with fine chromatin and visible nucleoli mimicking microgranular acute promyelocytic leukemia (A). Nucleated red blood cells were also present, consistent with myelophthisic anemia (C). The clinical/laboratory presentation, along with rouleaux formation (A, B), and plasmacytoid appearance raised suspicion for plasma cell leukemia. Flow cytometric analysis of peripheral blood showed plasma cells positive for CD38, CD138, and cLambda, and negative for CD19, CD45, CD56, and cKappa (E). No blasts were detected. Bone marrow examination confirmed 90% involvement by lambda‐restricted plasma cells with a complex karyotype showing four subclones and FISH positive for t(14;16)(q32;q23). This case highlights the variable morphology that can be seen in malignant plasma cells, in this case mimicking hypogranular acute promyelocytic leukemia.

## AUTHOR CONTRIBUTION

E.A. wrote the article, M.O.N. wrote the article and acquired the images, A.V.M. consented the patient and reviewed the article.

